# Nanosphere-in-a-nanoegg: damping the high-order modes induced by symmetry breaking

**DOI:** 10.1186/s11671-015-0728-3

**Published:** 2015-01-28

**Authors:** Jun Qian, Yi-Ding Sun, Yu-Dong Li, Jing-Jun Xu, Qian Sun

**Affiliations:** MOE Key Laboratory of Weak Light Nonlinear Photonics, Tianjin Key Laboratory of Photonics Material and Technology, School of Physics, Nankai University, Tianjin, 300071 China

**Keywords:** Surface plasmon resonance, Nanoshells, Mode suppression, Au-silica-Au, Plasmon hybridization, 78., 78.67.-n, 78.68. + m

## Abstract

We study the optical properties of the nanosphere-in-a-nanoegg structure (NSNE) by the three-dimensional finite difference time domain method. We demonstrate the suppression of the high-order plasmon modes in NSNE, which is induced by the plasmon interaction between the inner nanosphere and the outer nanoegg shell. A two-layer plasmon hybridization model is presented to explain this mechanism. The results we showed for plasmon mode suppression would be important to the design of the metal plasmonic devices. In addition, due to high tunable plasmon resonances in the near-infrared region (700 to 1,300 nm) with sub-100-nm size, NSNE can serve as a good substitute for the Au-silica-Au multilayer nanoshells in biological applications. Furthermore, compared with the Au-silica-Au nanoshells, NSNE has the advantage that the strong field enhancement can be achieved at the outer surface of the Au shell.

## Background

Gold (Au) nanoshells, which consist of a dielectric core surrounded by an ultrathin Au shell, have generated great interest at present due to their unique electronic and optical properties which are dominated by the localized surface plasmon resonance (LSPR). They have a broad range of spectroscopic and biomedical applications such as localized LSPR sensing [1], drug delivery [2], biomedical imaging [3], biological detection [4], and cancer therapeutics [5,6]. The plasmon resonances of Au nanoshells are greatly dependent on geometry and local dielectric environment. Recently, the Au-silica-Au multilayer nanoshells have been fabricated and widely studied [7-19]. Compared with the Au nanoshell, the Au-silica-Au nanoshells can provide more tunable plasmon resonance because of the plasmon mode interaction between the inner Au sphere and the outer Au shell, as explained by plasmon hybridization theory [20].

Breaking the symmetry of the Au nanoshell or Au-silica-Au multilayer nanoshells by dielectric (or Au) core offset can change the coupling behavior of plasmon modes [14,15,21-26]. In the plasmon hybridization theory, the plasmon resonance of the nanoshell can be considered as the interaction between plasmons of a sphere and a cavity [18]. In the spherically symmetric nanoshells, multipoles of the primitive plasmon modes can interact only with modes of the same order: dipolar sphere modes hybridize only with dipolar cavity modes, for example. For the nonconcentric geometries [15,21,22], the selection rules of plasmon interaction are relaxed, allowing modes of different orders to mix. The high-order plasmon modes are often excited in the spectrum of the nonconcentric structures. The symmetry breaking with nonconcentric geometry had been first studied in Au nanoegg by Halas's group [21]. With the increase of the silica core offset, multiple high-order plasmon resonance peaks appear in the spectrum of the nanoegg. These continuous and uneasily distinguishable high-order plasmon peaks in the spectra are unfavorable for some optical applications such as sensing. In the present work, we demonstrate that inserting an Au core into the nanoegg can suppress the high-order modes induced by symmetry breaking while maintaining the distinct dipolar plasmon modes. The results we showed are useful for manipulation of plasmon modes in plasmonic nanostructures.

For various biological applications of nanoshells, it is necessary to shift the plasmon resonance to the near-infrared region (700 to 1,300 nm) ‘biological window’ [27], which can be achieved by changing the ratio of the core size to shell thickness [28]. To obtain a strong plasmon resonance in the biological window, the size of the nanoshell should be larger than 150 nm [8]. But in some applications such as cancer therapy and contrast agents in biomedicine, nanoparticles in the sub-100-nm-size region are preferred, as they can easily penetrate the biological tissues. The Au-silica-Au multilayer nanoshells, an effective alternative to single nanoshells, can provide the strong plasmon resonance of $\left|{\omega}_{-}^{-}\right\rangle$ mode in the near-infrared region through plasmon hybridization, while maintaining the small size less than 100 nm [7,8]. However, in the $\left|{\omega}_{-}^{-}\right\rangle$ mode of the Au-silica-Au multilayer nanoshells, the strong near-field enhancement is almost confined inside the dielectric between the Au core and the outer Au shell [10,14], which is unfavorable for applications such as biosensing and bioimaging. The nanosphere-in-a-nanoegg (NSNE) structure we proposed can obtain the strong field enhancement at the outer surface of the Au shell in the plasmon mode of the near-infrared region, because the localized field penetrates through the thin part of the nonconcentric Au shell. The NSNE can be experimentally fabricated following the reported anisotropic electroless plating technique [21].

In this letter, we investigate the optical properties of NSNE by the three-dimensional finite difference time domain (FDTD) method. We demonstrate that inserting a symmetric metal structure (nanosphere) into the unsymmetric metal structure (nanoegg) can suppress the high-order modes induced by symmetry breaking. The results we showed would be important to the design of the metal plasmonic devices. A two-layer plasmon hybridization is presented to explain the physical mechanism of plasmon mode suppression [29]. We show that NSNE could be a good alternative of Au-silica-Au multilayer nanoshells due to its highly tunable plasmon resonances in the near-infrared region (700 to 1,300 nm) with sub-100-nm size. Furthermore, compared with the Au-silica-Au structure, NSNE provides the advantage that the strong field enhancement can be achieved at the outer surface of the Au shell, which is very useful for light-based applications such as biomedical imaging and sensing [1,3].

## Methods

**Figure 1:**
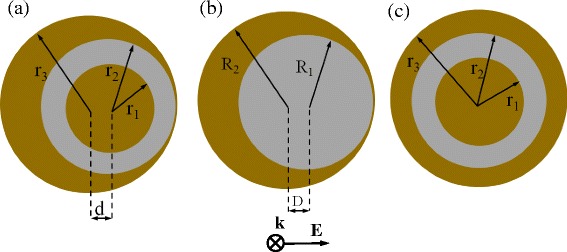
**Mid-sectional view and the structure parameters of NSNE (a), the nanoegg (b), and Au-silica-Au multilayer structure (c).** The polarization (*E*) and propagation (*k*) directions of incident light are denoted.

The schematic geometry and the structure parameters of NSNE are shown in Figure 1a. For comparison, the nanoegg and the Au-silica-Au structure are also shown in Figure 1. The radii of each layer of NSNE and Au-silica-Au structure are *r*_1_, *r*_2_, and *r*_3_. The core offset of NSNE is *d*. The radii of the silica core and Au shell of nanoegg are *R*_1_ and *R*_2_, and the core offset is *D*. A total-field scattering-field light source with an electric field polarization denoted in Figure 1 is used in the FDTD simulations. The dielectric constant of the silica layer is set as 2.04. The current literature on the surface scattering effect [30] of the core-shell nanoshells has not been able to reach a consensus. It was reported that the surface scattering has little impact on the resonance line widths [31]. However, others propose that the effect of the surface scattering on the resonance line widths is determined by the core radius to shell thickness ratio [32]. In our simulations, for simplicity, the experimentally measured dielectric function is utilized for gold shell [33], neglecting the electron surface scattering effect.

## Results and discussion

**Figure 2:**
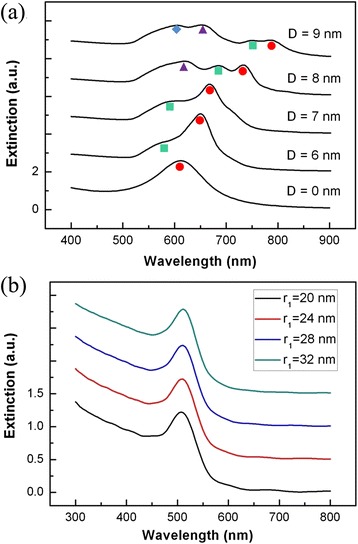
**The extinction spectrum of the nanoegg and the Au sphere. (a)** The multipolar extinction spectra of the nanoegg with different core offset *D* at *R*_1_/*R*_2_ = 36 nm/46 nm. Various colored shapes indicate the peak wavelengths of multipolar extinction spectra (red circles, *l* = 1; green squares, *l* = 2; purple triangles, *l* = 3; blue diamond, *l* = 4). **(b)** The extinction spectrum of the Au sphere with different radius *r*_1_.

Figure 2a shows the extinction spectrum of the nanoegg with different core offset *D* at *R*_1_/*R*_2_ = 36 nm/46 nm. In the text, *l* is the multipolar index (dipole, *l* = 1; quadrupole, *l* = 2…). For the core offset *D* = 0 nm (nanoshell), there is only a dipolar mode (red circles, *l* = 1) in the spectrum. As the core offset *D* increases, several high-order plasmon resonance modes appear. At the core offset *D* = 9 nm, there are four plasmon resonance modes (*l* = 1, 2, 3, 4) in the spectrum. All orders of modes show redshift with the increase in the core offset *D*. The results agree with the previous study of nanoegg [21,22]. Figure 2b shows the extinction spectrum of the Au sphere with different radius *r*_1_. The extinction peaks slightly redshift with increased radius *r*_1_.

**Figure 3:**
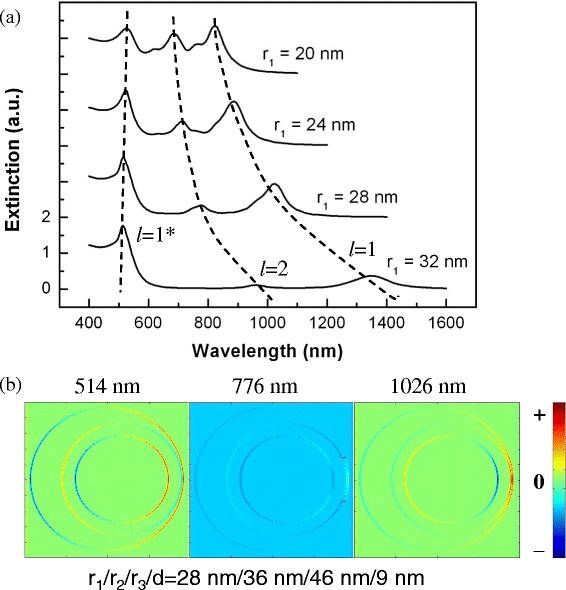
**The extinction spectra and charge density distributions. (a)** The extinction spectra of NSNE with different radius of Au core *r*_1_ at *r*_2_/*r*_3_ = 36 nm/46 nm and *d* = 9 nm. The dashed line indicates the high-energy dipolar mode (*l* = 1*), the quadrupolar mode (*l* = 2), and the low-energy dipolar mode (*l* = 1). **(b)** The charge density distributions of the high-energy dipolar mode (${\left|{\omega}_{-}^{+}\right\rangle}_1$, 514 nm), the quadrupolar mode (${\left|{\omega}_{-}^{-}\right\rangle}_2$, 776 nm), and the low-energy dipolar mode (${\left|{\omega}_{-}^{-}\right\rangle}_1$, 1,025 nm) of NSNE with *r*_1_/*r*_2_/*r*_3_/*d* = 28 nm/36 nm/46 nm/9 nm.

In order to damp the high-order modes of the nanoegg with large core offset, we insert an Au sphere core into the nanoegg with the core offset *D* = 9 nm at *R*_1_/*R*_2_ = 36 nm/46 nm. Figure 3 shows the extinction spectrum of NSNE with different radius of Au core *r*_1_ at *r*_2_/*r*_3_ = 36 nm/46 nm and *d* = 9 nm. It is found that, with the increase of the *r*_1_, most high-order modes are weakened and finally disappear in the spectrum. When *r*_1_ is larger than 24 nm, there are only three plasmon modes (the high-energy dipolar mode, *l* = 1*; the quadrupolar mode, *l* = 2; the low-energy dipolar mode, *l* = 1) in the spectra. The details of the mode coupling are described in the following paragraphs. The charge density distributions of these plasmon modes for the radius of Au core *r*_1_ = 28 nm are calculated. As shown in Figure 3b, the plasmon mode at 514 and 1,026 nm are dipolar modes, and the plasmon mode at 776 nm is a quadrupolar mode. Moreover, as shown in Figure 3, the low-energy dipolar modes show distinct redshift with the increase of *r*_1_, and its wavelength shifts from 800 to 1,300 nm, which covers the wavelength range of the biological window.

**Figure 4:**
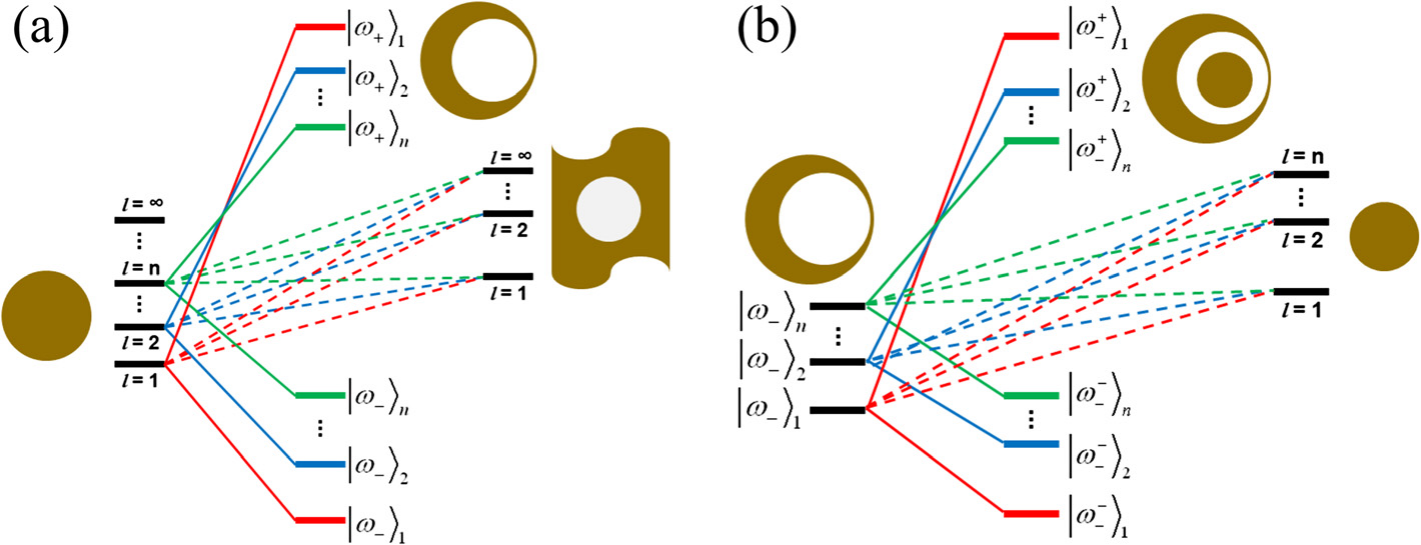
**An energy-level diagram describing the plasmon hybridization in the nanoegg (a) and in NSNE (b).**

The suppression of the high-order modes can be explained by the two-layer plasmon hybridization model. In the plasmon hybridization theory, the plasmon resonances of NSNE can be considered as the interaction between plasmon modes of a nanoegg and a sphere. Further, the plasmon resonances of the nanoegg can be viewed as the interaction between plasmon modes of a sphere and a cavity. Figure 4a shows the plasmon hybridization picture of a nanoegg. In the nonconcentric geometries [15,21,22], the plasmon interaction between different orders of plasmon modes is allowed. The interaction creates the low-energy bonding modes (|*ω*_−_〉_1_, |*ω*_−_〉_2_, …, |*ω*_−_〉_*n*_) and the high-energy antibonding modes (|*ω*_+_〉_1_, |*ω*_+_〉_2_, …, |*ω*_+_〉_*n*_) of different orders. The high-order antibonding modes (|*ω*_+_〉_*n*_, *n* ≥ 2) are always weak and unvisualized in the spectrum [8,21]. In Figure 2, the bonding modes (|*ω*_−_〉_*n*_) of nanoegg with different core offset *D* are shown. With the increase of core offset *D*, the plasmons of the core interact more strongly with the plasmons of the shell. These increasing interactions lead to stronger mixing between the different modes, causing a large redshift of the bonding modes, which is also seen in other symmetry-breaking nanostructures [17,34]. The |*ω*_+_〉_1_ modes (at about 300 nm) are not presented in Figure 2.

Figure 4b shows the plasmon hybridization picture of NSNE. The plasmon resonances of NSNE are mainly considered as the interaction between the low-energy bonding modes (|*ω*_−_〉_1_, |*ω*_−_〉_2_, …, |*ω*_−_〉_*n*_) of the nanoegg and the sphere (the interaction between the high-energy |*ω*_+_〉_1_ mode of the nanoegg and the sphere is very weak and not considered). The interaction creates the low-energy hybridized bonding modes (${\left|{\omega}_{-}^{-}\right\rangle}_1$, ${\left|{\omega}_{-}^{-}\right\rangle}_2$, …, ${\left|{\omega}_{-}^{-}\right\rangle}_n$) and the high-energy hybridized antibonding modes (${\left|{\omega}_{-}^{+}\right\rangle}_1$, ${\left|{\omega}_{-}^{+}\right\rangle}_2$, …, ${\left|{\omega}_{-}^{+}\right\rangle}_n$) of different orders. The high-order (${\left|{\omega}_{-}^{+}\right\rangle}_n$, *n* ≥ 2) hybridized antibonding modes are weak and unvisualized in the spectrum. Due to the nonconcentric geometry of the nanoegg, the different orders of plasmon modes between the nanoegg and the sphere are also allowed to be mixed. However, as a nanosphere is inserted into the center of the nanoegg cavity, because of the strong interaction between the sphere and the nanoegg [22], the plasmon interaction between different orders of plasmon modes are mostly weakened. Under this interaction, the strength of the high-order (${\left|{\omega}_{-}^{-}\right\rangle}_n$, *n* ≥ 3) hybridized bonding modes becomes very small. Only the dipolar and quadrupolar bonding modes (${\left|{\omega}_{-}^{-}\right\rangle}_n$, *n* = 1, 2) are maintained in the spectrum. In Figure 3, with the increase of the *r*_1_, it is seen that only the dipolar bonding mode, the quadrupolar bonding mode, and the dipolar antibonding mode are visualized in the spectrum. For example, at *r*_1_ = 28 nm, the dipolar bonding mode (${\left|{\omega}_{-}^{-}\right\rangle}_1$), the quadrupolar bonding mode (${\left|{\omega}_{-}^{-}\right\rangle}_2$), and the dipolar antibonding mode (${\left|{\omega}_{-}^{+}\right\rangle}_1$) are located at 1,026, 776, and 514 nm, respectively. The dipolar bonding mode (${\left|{\omega}_{-}^{-}\right\rangle}_1$) and the dipolar antibonding mode (${\left|{\omega}_{-}^{+}\right\rangle}_1$) are similar to the corresponding modes in the Au-silica-Au multilayer nanoshells [35]. From the charge distribution of the quadrupolar bonding mode, we can find that the quadrupolar mode of the core and nanoegg has a dipole component, which indicates a mixed hybridization in NSNE. Moreover, an increase in the *r*_1_ will slightly decrease the energy of the Au sphere mode (redshift as shown in Figure 2b), which may decrease the energy of the hybridized bonding and antibonding modes. On the other hand, an increased *r*_1_ will reduce the thickness of the middle silica layer and increase the plasmon interaction between the Au core and the Au shell, which can induce the energy enhancement of the antibonding modes and the energy reduction of bonding modes. The influence of the enhanced coupling between the Au core and Au shell is much stronger than that due to energy reduction of the Au sphere mode. Thus, we can see the redshift of the dipolar and quadrupolar bonding modes and the slight blueshift of the dipolar antibonding mode with the increase of *r*_1_ in Figure 3.

**Figure 5:**
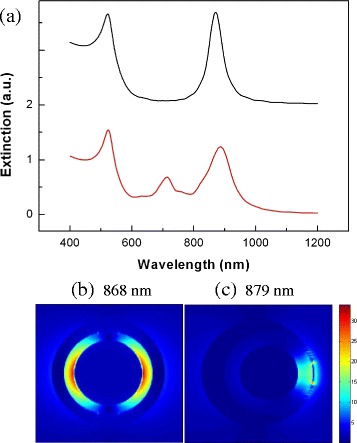
**The extinction spectrum and near-field distributions. (a)** The extinction spectrum of the Au-silica-Au multilayer nanoshells with *r*_1_/*r*_2_/*r*_3_ = 28 nm/36 nm/46 nm in black line and the extinction spectrum of NSNE with *r*_1_/*r*_2_/*r*_3_/*d* = 24 nm/36 nm/46 nm/9 nm in red line. The near-field distributions of the ${\left|{\omega}_{-}^{-}\right\rangle}_1$ mode of the Au-silica-Au multilayer nanoshells at 868 nm **(b)** and NSNE at 879 nm **(c)**.

We compare the optical properties of the Au-silica-Au multilayer nanoshells and NSNE in Figure 5. For example, the extinction spectrum of the Au-silica-Au multilayer nanoshells with *r*_1_/*r*_2_/*r*_3_ = 28 nm/36 nm/46 nm is shown in black line in Figure 5a. The plasmon resonance of the ${\left|{\omega}_{-}^{-}\right\rangle}_1$ mode (at 868 nm) is useful for biological applications in the biological window. The near-field distributions of the ${\left|{\omega}_{-}^{-}\right\rangle}_1$ mode are shown in Figure 5b. It is seen that the strong field enhancement (here defined as *E*_s_ = |*E*|/|*E*_0_| where *E*_0_ is the electric field of the illumination wave) is almost confined inside the dielectric layer between the Au core and the outer Au shell. The local field is symmetrically distributed on both sides of the dielectric layer. Because the local field distributions of the multilayer nanoshells is sensitive to the wavelength of plasmon resonance peak [9], in order to compare the field distributions of ${\left|{\omega}_{-}^{-}\right\rangle}_1$ modes in NSNE with that of ${\left|{\omega}_{-}^{-}\right\rangle}_1$ modes at 868 nm in the Au-silica-Au multilayer nanoshells, we choose NSNE with geometric parameters *r*_1_/*r*_2_/*r*_3_/*d* = 24 nm/36 nm/46 nm/9 nm, which has a similar wavelength (at 879 nm) of ${\left|{\omega}_{-}^{-}\right\rangle}_1$ modes. The extinction spectrum of NSNE is shown in red line in Figure 5a. The near-field distributions of the ${\left|{\omega}_{-}^{-}\right\rangle}_1$ mode (at 879 nm) of NSNE are shown in Figure 5c. It is found that the strong field enhancement is localized at the dielectric layer near the thin part of the nonconcentric Au shell. The localized field penetrates through the thin part of the Au shell, so the large field enhancement is obtained at the outer surface of the Au shell. The maximum field enhancement at the outer surface of the Au shell in NSNE is *E*_s-max_ = 14.15, whereas that of the Au-silica-Au nanoshells is *E*_s-max_ = 5.08. Compared with the Au-silica-Au nanoshells, nearly threefold improvement of the *E*_s-max_ outside the Au shell is achieved in NSNE.

**Figure 6:**
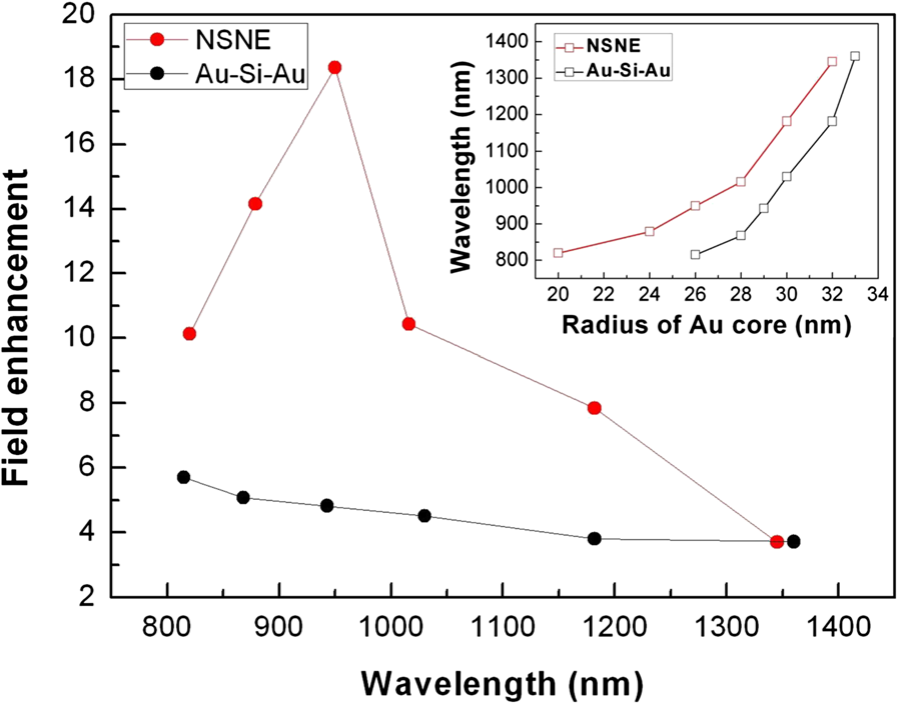
**Maximum field enhancements** ***E***_s-max_ **.** The maximum field enhancements *E*_s-max_ outside the Au shell of the Au-silica-Au multilayer nanoshells (black dots) and NSNE (red dots) at different resonant wavelengths of ${\left|{\omega}_{-}^{-}\right\rangle}_1$ modes. Inset: dependence of the resonant wavelength of ${\left|{\omega}_{-}^{-}\right\rangle}_1$ modes on the Au core radius *r*_1_ in the Au-silica-Au multilayer nanoshells (black squares) and NSNE (red squares).

In Figure 6, the maximum field enhancements *E*_s-max_ outside the Au shell of the Au-silica-Au multilayer nanoshells and NSNE at different resonant wavelengths of ${\left|{\omega}_{-}^{-}\right\rangle}_1$ modes are compared. It is seen that the *E*_s-max_ of the Au-silica-Au multilayer nanoshells slightly decreases with the increase of resonant wavelength, whereas in NSNE, the *E*_s-max_ first increases and then decreases with the increase of resonant wavelength. The maximum *E*_s-max_ (*E*_s-max_ = 18.36) of NSNE is obtained at 950 nm. In the most wavelength range (800 to 1,200 nm) of biological windows, the *E*_s-max_ of NSNE is obviously larger than that of the Au-silica-Au nanoshells. At the large wavelength (>1,300 nm), the *E*_s-max_ of NSNE is approximately equal to that of the Au-silica-Au nanoshells. In order to get the similar wavelength of ${\left|{\omega}_{-}^{-}\right\rangle}_1$ modes, different Au core radius *r*_1_ of NSNE and the Au-silica-Au nanoshells is selected, while the *r*_2_ and *r*_3_ of NSNE and the Au-silica-Au nanoshells are fixed at 36 and 46 nm. In the inset of Figure 6, the dependences of the resonant wavelength of ${\left|{\omega}_{-}^{-}\right\rangle}_1$ modes on the Au core radius *r*_1_ are shown for the Au-silica-Au nanoshells and NSNE.

Recently, the Fano resonances have been investigated in the Au-silica-Au multilayer nanoshells [15-19]. It is demonstrated that the tunable Fano resonance can be achieved in the symmetric [15] or unsymmetric (Au core offset) [16] Au-silica-Au multilayer nanoshells by adjusting the geometric parameters. In the NSNE, with the increase of *r*_1_, the interaction of the Au core and the nanoegg shell is increased, and the split of the dipolar antibonding mode and the dipolar and the quadrupolar bonding mode is enhanced, which avoids the overlap of the plasmon peaks in the spectra. Thus, the Fano resonances have not been found in the NSNE systems.

**Figure 7:**
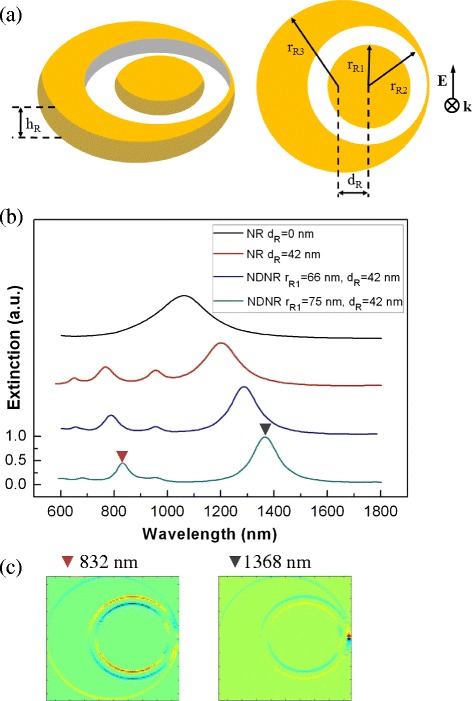
**The structure parameters and extinction of the concentric nanoring. (a)** The structure parameters of the NDNR. The polarization (*E*) and propagation (*k*) directions of incident light are denoted. **(b)** The extinction of the concentric nanoring with *r*_R1_/*r*_R2_/*r*_R3_/*d*_R_ = 0 nm/90 nm/135/0 nm (black line), asymmetric nanoring with *r*_R1_/*r*_R2_/*r*_R3_/*d*_R_ = 0 nm/90 nm/135 nm/42 nm (red line), the NDNR with *r*_R1_/*r*_R2_/*r*_R3_/*d*_R_ = 66 nm/90 nm/135 nm/42 nm (blue line), and the NDNR with *r*_R1_/*r*_R2_/*r*_R3_/*d*_R_ = 75 nm/90 nm/135 nm/42 nm (cyan line). **(c)** The charge density distributions of the dipolar antibonding mode (832 nm) and the dipolar bonding mode (1,368 nm) of the NDNR with *r*_R1_/*r*_R2_/*r*_R3_/*d*_R_ = 75 nm/90 nm/135 nm/42 nm.

To further confirm the idea that inserting a symmetric metal structure into the unsymmetric metal structure can suppress the high-order modes, we investigate the asymmetric Au nanoring structures. The symmetric and asymmetric nanoring structures have been well studied in recent years [34,36]. The nanodisk-in-an-asymmetric-nanoring (NDNR) structure is shown in Figure 7a. The asymmetric nanoring is defined by four parameters, the inner radius *r*_R2_, the outer radius *r*_R3_, the offset of the centers *d*_R_, and the height *h*_R_. The radius of the inserted nanodisk is *r*_R1_. The height of the system *h*_R_ is 42 nm. Figure 2b shows the extinction of the concentric nanoring with *r*_R1_/*r*_R2_/*r*_R3_/*d*_R_ = 0 nm/90 nm/135 nm/0 nm, asymmetric nanoring with *r*_R1_/*r*_R2_/*r*_R3_/*d*_R_ = 0 nm/90 nm/135 nm/42 nm, and the NDNR with *r*_R1_ = 66 and 75 nm at other parameters *r*_R2_/*r*_R3_/*d*_R_ = 90 nm/135 nm/42 nm. It is seen that, when the offset *d*_R_ is increased, the multipolar modes of nanoring appear in the spectrum. Furthermore, inserting a nanodisk into the asymmetric nanoring can suppress the high-order modes, and only the dipolar modes remain in the spectra, as shown in Figure 7b,c. Similar to the NSNE structure, the strong interaction between the nanodisk plasmons and the nanoring plasmons leads to this mode damping effect.

## Conclusions

In summary, we theoretically studied the optical properties of NSNE. We demonstrated that the multiple high-order modes induced in nanoegg by symmetry breaking can be suppressed by inserting an Au sphere into its cavity. A two-layer plasmon hybridization model is presented to explain this process. The wavelength of bonding mode of NSNE is sensitive to the Au core radius *r*_1_ and can be tuned in the near-infrared region (700 to 1,300 nm) that is suited to the biological applications. Due to the high tunability of plasmon resonance in the biological window with sub-100-nm size, NSNE could be a good alternative of the Au-silica-Au multilayer nanoshells and provide the further advantage that the strong field enhancement can be achieved at the outer surface of the Au shell.

## Abbreviations

LSPR: localized surface plasmon resonance
NSNE: nanosphere-in-a-nanoegg
NDNR: nanodisk-in-an-asymmetric-nanoring
FDTD: finite difference time domain
